# Some Scientific Aspects of Insanity

**Published:** 1891-12-12

**Authors:** 


### Some Scientific Aspects of Insanity.

### VI.—EARLY STAGES.

In the preceding pages I have spoken of insanity as a failure on the part of a nervous system to adapt itself to its surroundings. Each animal in accordance to its intelligence is able, when in perfect health, to live in harmony with its environment. It avoids extremes of heat or extremes of cold; it finds its appropriate food when it is hungry; it rests when exhausted and fatigued, and it defends itself from its enemies either by flight or resistance. As we ascend in the animal scale, the circumstances to which each successive higher grade has to adapt itself become more and more complicated. An animal such as a dog has merely to observe the laws of self-preservation and any additional rules that may be imposed by domestic living. The circumstances of life with which a man is surrounded on the other hand are infinitely more complex. Not only has he, like the dog, to respect the cruder laws of nature, but he has also to accord with the laws and conventionalities of his fellow-men, and more especially of the society in which he is situated. Again, there is a vast difference in complexity of surrounding circumstances between the life of a savage and the life of a highly-civilised man. While the savage has only to regard the simpler rules of his society, a civilised man has to adapt himself to such extremely subtle circumstances as changes in public opinion, the rise and fall of stocks, questions of political economy, and the latest results of science.

A failure on the part of any person to adjust himself to the customs and manners of his society is called eccentricity. A failure on the part of any person to adjust himself to the standard of conduct of his society is known as immorality; and a failure of adjustment to the intelligence, or to the conduct, or to the intelligence and conduct combined, of the society, is termed insanity. With advancing civilisation the complexity of the surroundings is always increasing, and at the same time the standard of every progressive society is being raised, so that it is always becoming more difficult for those people whose nervous system is ill adapted for the battle of life to rise with the standard. Again, society is more intolerant of people of eccentric habits, of people who openly express delusions, or of people who see prophetic visions and recount them.

The patients who form the great bulk of the inmates of modern lunatic asylums are, therefore, persons who have failed to adjust themselves to their surroundings for one or other of the following reasons: 1. They may be mentally depressed to such an extent as to make them actively desirous of ending their own existence, or they may be affected by lowness of spirits in such a way as to render them unfit to follow their ordinary avocations. 2. They may be mentally exalted to such a degree as to render their excitement offensive to others; or they may be aggressive and dangerous to their fellows. 3. They may be the subjects of fixed delusions. 4. They may suffer from some of the distinctive forms of insanity, associated either with convulsions or paralysis, or both. 5. They may be the subjects of dementia (loss of mental power), either primary or secondary.

I. *States of Mental Depression.*—Of all mental diseases melancholia is the one least removed from sanity, because many sane men suffer from mental depression very acutely, and because it is not necessarily accompanied by any intellectual disturbance. Melancholia may be defined as mental pain in the same way as neuralgia may be defined as bodily pain. Now, neuralgia may have a well-known cause, such as decay of a tooth. When the tooth is extracted the pain ceases. But there may be neuralgia without any apparent cause, which lasts indefinitely, and which every remedy fails to cure. In the same way there may be melancholy due to privation, loss of friends, or loss of wealth, which passes away naturally within a reasonably short time, or it is removed immediately should its cause be removed. This form of melancholy is looked upon as natural, but when depression of spirits comes on without any apparent cause, when it lasts indefinitely, and when it resists all attempts to remove it, it is called melancholia.

The word "melancholia" is derived from a Greek adjective and noun—*melan*, black, and *cholē*, bile. The dark pigmentation of the skin in cases labouring under this disease is very frequently met with. The face is usually pale, the features drawn out, and the expression distressed or distracted. The victim stoops as if his miseries were weighing heavily upon him. His muscles are flabby, and there is a prominent lack of that energy and tone which characterises the movements of the body in health. The manner of the patient is listless and apathetic. He pays very little attention to what is going on around him; all his interest is absorbed in meditation upon his own sufferings. If his misery is more intense, he becomes restless, wrings his hands, moves about rapidly through the room, he becomes still more engrossed in himself, and pays less and less attention to his surroundings. There is yet another stage of intensity in which the patient is completely overborne by the disease, in which the feeling of pain is so great that it completely stuns the patient, and he passes into a state resembling stupor.

